# Proteomic Profiling of Limited-Stage Follicular Lymphoma Reveals Differentially Expressed Proteins Linked to Disease Progression Post-Radiation Therapy

**DOI:** 10.3390/ijms26199306

**Published:** 2025-09-23

**Authors:** Jonas Klejs Hemmingsen, Marie Hairing Enemark, Emma Frasez Sørensen, Cecilie Dohrmann, Kristina Lystlund Lauridsen, Stephen Jacques Hamilton-Dutoit, Robert Kridel, Bent Honoré, Maja Ludvigsen

**Affiliations:** 1 Department of Hematology, Aarhus University Hospital, 8200 Aarhus N, Denmark; 2 Department of Clinical Medicine, Aarhus University, 8200 Aarhus N, Denmark; 3 Department of Pathology, Aarhus University Hospital, 8200 Aarhus N, Denmark; 4 Princess Margaret Cancer Centre, University Health Network, Toronto, ON M5G 2M9, Canada; 5 Department of Biomedicine, Aarhus University, 8000 Aarhus C, Denmark

**Keywords:** non-Hodgkin lymphoma, follicular lymphoma, disease progression, proteomics, radiation therapy

## Abstract

Follicular lymphoma (FL) is the most common indolent lymphoma. Despite a generally favorable prognosis and long-term survival for many patients, FL remains incurable, with disease progression occurring in approximately half of limited-stage FL cases. In this study, we employed high-throughput mass spectrometry-based proteomics to explore the differential protein expression in diagnostic lymphoma biopsies from 26 limited-stage FL patients. Of these, 9 patients experienced subsequent disease progression (sp-FL), while 17 did not (np-FL). A total of 1940 proteins were identified, with 78 showing significant differential expression between progressing and non-progressing cases. Unsupervised clustering analyses were able to separate the two patient groups based on these differential protein profiles. Notably, proteins involved in metabolism, immune regulation, and apoptosis were downregulated in sp-FL samples. Among the identified proteins, caspase 4 and 8 (CASP4 and CASP8) were further evaluated. The low expression of CASP4 in the diagnostic lymphoma tissue correlated with shorter progression-free survival (PFS) (*p* < 0.001), primarily with this difference apparent in the expression profiles in the intrafollicular areas (*p* = 0.015). Similarly, low CASP8 expression was associated with inferior PFS (*p* = 0.031). Interestingly, addressing the expression pattern for advanced-stage FL patients, the low protein expression of both CASP4 and CASP8 was also found to be associated with progressing cases, suggesting their potential role in disease pathogenesis independent of the disease stage. With further research, the expression pattern of CASP4 and CASP8 may enable the early prediction of disease progression in FL patients, which may ultimately improve patient stratification and allow for more individualized treatment strategies.

## 1. Introduction

Follicular lymphoma (FL) is the second most prevalent non-Hodgkin lymphoma in Western countries, constituting around 20% of all lymphomas [1,2]. FL is characterized by the malignant expansion of B cells, typically harboring the t(14:18) translocation [3,4]. This translocation results in a constitutive transcription and expression of the anti-apoptotic B-cell lymphoma 2 protein, BCL2, enhancing B-cell survival. While this translocation is a hallmark of FL, additional alterations are essential for a malignant development; currently however, these alterations are not clearly defined [5].

The prognosis of FL is generally favorable, as demonstrated by the survival rates measured in decades [6]. FL patients exhibit highly heterogeneous clinical trajectories, but FL is still considered an incurable malignancy [5,7]. This clinical heterogeneity is reflected in standard therapeutic regimens where the choice of treatment largely depends on the disease stage. Management approaches range from a “watch and wait” strategy or radiation therapy to more aggressive regimens including the combination of rituximab with chemotherapy [6,8]. Patients diagnosed with limited-stage FL, i.e., stages I and II, according to the Ann Arbor staging system, often exhibit favorable responses to initial therapy, with the majority of patients achieving prolonged remissions [9,10]. For this patient group, treatment may even be administered with curative intent. However, roughly one half of these patients experience disease progression, ultimately impacting their prognosis and thus overall survival [11,12,13]. Identifying prognostic biomarkers for progression risk in this patient group remains a critical unmet need to optimize therapeutic strategies and improve patient outcomes [14].

The underlying FL tumor biology driving disease progression remains unresolved despite substantial efforts to enable prediction of the clinical course of the disease [15]. To clarify the undisclosed aspects of FL and improve our understanding of FL progression, we conducted an exploratory investigation using large-scale, mass spectrometry (MS)-based proteomics. Diagnostic lymphoma biopsy samples from patients with limited-stage FL, all treated with radiation therapy, were analyzed for protein expression to identify novel predictive biomarkers of progression.

## 2. Results

### 2.1. Patient Characteristics

The patient cohort comprised 26 limited-stage FL patients treated with radiation therapy. Hereof, 9 patients experienced subsequent disease progression, while 17 patients remained without progression (Table 1 and Figure S1). The study included 10 males and 16 females with a median age at diagnosis of 69 years (range of 23–86 years). There were no significant differences in the common clinicopathological features between the sp-FL and np-FL samples at the time of the initial diagnosis. The median time to progression was 5.25 years (range of 0.34–14.12 years).

### 2.2. Proteomic Profiling Identifies Differentially Expressed Proteins Correlated to Subsequent Disease Progression

**Figure 1:**
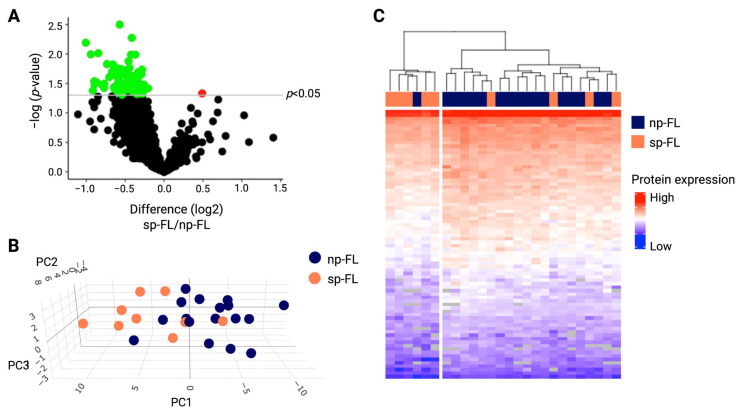
Differentially expressed proteins between np-FL and sp-FL samples identified by MS-based proteomics. (**A**) Volcano plot depicting all 1940 proteins identified in diagnostic np-FL and sp-FL samples. The *x*-axis represents fold changes (transformed by log_2_; sp-FL/np-FL), and the *y*-axis shows the corresponding *p*-value (transformed by log_10_), with the gray horizontal line marking *p*-values below 0.05. Thus, red dots mark significantly upregulated proteins, while green dots mark significantly downregulated proteins in the subsequently progressing group. (**B**) 3D PCA plot with input of significantly differentially expressed proteins between np-FL and sp-FL samples (67 proteins identified without missing values). (**C**) Heatmap and hierarchical clustering with input of the identified 78 significantly differentially expressed proteins. Each row represents one protein, while each column represents one sample. Abbreviations: PC, principal component; np-FL, non-progressing FL; and sp-FL, subsequently progressing FL.

From the nLC-MS/MS, a total of 1940 proteins were identified across the 26 samples, including 78 significantly differentially expressed proteins between np-FL and sp-FL samples (Table S1 and Figure 1A). Interestingly, we observed the general significant downregulation of protein expression in sp-FL samples, with 77 of the 78 significantly differentially expressed proteins being downregulated (fold changes 0.50–0.87) and only one upregulated (fold change 1.40) in the sp-FL samples. Among the 77 downregulated were several metabolism-related proteins including triosephosphate isomerase 1 and enolase 1 (ENO1), as well as apoptotic and immune regulators caspase 4 and 8 (CASP4, CASP8), Janus kinase 1 (JAK1), and mitogen-activated protein kinase 14 (MAPK14).

To assess if the protein expression levels could distinguish FL samples based on their risk of subsequent progression, an unsupervised PCA and hierarchical clustering were conducted. Based on input from all identified proteins, no noticeable distinctions were observed (Figure S2). However, based on the significantly differentially expressed proteins as input, the analysis revealed a pattern showing focused clustering corresponding to either np-FL or sp-FL groups, with a few samples intermingled. This suggests potential risk subgroups of FL tumors that are predictive of progression based on various protein expression levels (Figure 1B). This tendency was also evident when performing hierarchical clustering (Figure 1C). Here, samples were clustered into two main groups: a high-risk group including five sp-FL samples and only one np-FL sample, and a low-risk group comprising the remaining four sp-FL samples and sixteen np-FL samples. Notably, the one np-FL sample clustering in the high-risk group was a younger male with more than 10 years of follow-up, whose clinicopathological data showed no clear explanation for this grouping. In the low-risk group, the four sp-FL patients experienced progression with very different time points ranging from 0.3 to 14 years.

### 2.3. Differentially Expressed Proteins Suggest Alterations Within Metabolism and the Innate Immune System

To investigate the possible biological impact of differences in protein expression on important cellular pathways and the processes associated with disease progression, a gene enrichment analysis of the 78 significantly differentially expressed proteins was computed via the STRING database. The protein network consisted of 78 nodes and 421 edges, indicating more interactions than what would be expected at random (*p* < 0.001) (Table S2 and Figure S3). Within this network, distinct clusters of proteins were identified among the disrupted pathways, with several proteins participating in multiple processes, suggesting a link between cellular functions. The most evident alterations were observed in the processes related to metabolism (based on 26 proteins), RNA binding (based on 22 proteins), and NOD-like receptor signaling (based on 7 proteins). Notably, these disturbances were very similar to identified aberrancies in a previous study by our group investigating disease progression in advanced-stage FL patients [16], indicating that the progression of disease, independent of the disease stage, might be related to alterations of the metabolism and the innate immune system in the lymphomas [16].

### 2.4. Low Caspase 4 and Caspase 8 Correlates to Risk of Disease Progression in Limited-Stage FL

In a large-scale, MS-based study, several immune and apoptotic-related proteins were identified as significantly differentially expressed, including the two caspase proteins, CASP4 and CASP8. Both proteins were further evaluated by immunohistochemistry (IHC) in the lymphoma tissues. The IHC staining results were quantified through digital image analysis, with the quantified staining represented as area fractions (AFs), calculated as the ratio of the stained area to the total area within the region of interest (ROI) covering the neoplastic tissue.

**Figure 2:**
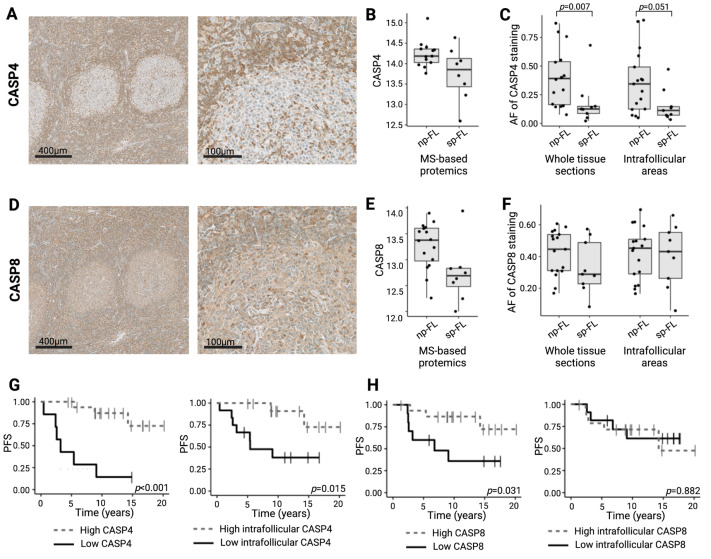
Immunohistochemical evaluation of CASP4 and CASP8 in limited-stage disease. (**A**) Representative images of immunohistochemical staining patterns of CASP4 expression (left, original magnification ×5; right, original magnification ×20). (**B**) CASP4 expression levels identified by MS-based proteomics. (**C**) CASP4 expression levels identified by immunohistochemical staining in whole biopsy and intrafollicular areas. (**D**) Representative images of immunohistochemical staining patterns of CASP8 expression (left, original magnification ×5; right, original magnification ×20). (**E**) CASP8 expression levels identified by MS-based proteomics. (**F**) CASP8 expression levels identified by immunohistochemical staining in whole biopsy and intrafollicular areas. (**G**) Association between whole biopsy and intrafollicular CASP4 expression and PFS (cutoffs, AF = 0.141 and AF = 0.146, respectively). (**H**) Association between whole biopsy and intrafollicular CASP8 expression and PFS (cutoffs, AF = 0.31 and AF = 0.4308, respectively). Abbreviations: AF, area fraction; CASP4/8, caspase 4/8; np-FL, non-progressing FL; OS, overall survival; PFS, progression-free survival; and sp-FL, subsequently progressing FL.

CASP4 displayed a strong cytoplasmatic expression in cells within both intrafollicular regions and in the tumor microenvironment, with staining generally weaker inside the follicles than in the surrounding interfollicular areas (Figure 2A). Similarly, CASP8 also showed a cytoplasmatic expression pattern with positive cells both within and between follicles (Figure 2D). In the MS analysis, both CASP4 and CASP8 were downregulated in sp-FL samples. When evaluated by IHC, CASP4 retained this significant difference in protein expression (*p* = 0.007). This difference was also observed when analyzed exclusively in intrafollicular areas, although without reaching the significance levels (*p* = 0.051) (Figure 2B,C). In contrast, the IHC evaluation of CASP8 showed no significant differences between sp-FL and np-FL samples. This was also evident when evaluating only the intrafollicular areas (*p* = 0.164 and *p* = 0.916, respectively) (Figure 2E,F).

To further understand the relationships between the two caspase proteins and various clinical parameters, correlations between the markers and clinicopathological data were analyzed. Expression levels of CASP4 and CASP8 showed a significantly strong positive correlation with each other (*p* < 0.001, ρ = 0.64; intrafollicular levels exclusively *p* = 0.018, ρ = 0.47), highlighting distinct patterns of association, suggesting potential coherent roles for these proteins in disease progression. Neither of the two proteins correlated to the clinicopathological parameters analyzed.

Low levels of CASP4 expression at the time of the initial FL diagnosis were associated with a significantly shorter progression-free survival (PFS) and also when evaluated exclusively in intrafollicular areas (*p* < 0.001 and *p* = 0.015, respectively) (Figure 2G). The same was observed for CASP8, with a low expression being associated with an inferior PFS when evaluating the protein expression throughout in the tumor tissue (*p* = 0.031) (Figure 2H). Notably, from the entire cohort, only five samples had concurrent low expressions of both CASP4 and CASP8. Four of these were sp-FL samples, while only one was an np-FL sample.

### 2.5. CASP4 and CASP8 Trend Towards an Implication in Disease Progression in Advanced-Stage FL

We previously performed an MS-based proteomics study investigating disease progression in a cohort of advanced-stage FL patients (Ann Arbor stages III-IV) [16]. Consistent with the findings of the present study, CASP4 was significantly downregulated in sp-FL samples compared with np-FL samples (*p* = 0.038, fold change 0.77). CASP8 was also identified in the previous study but did not show a differential expression between patient groups [16].

**Figure 3:**
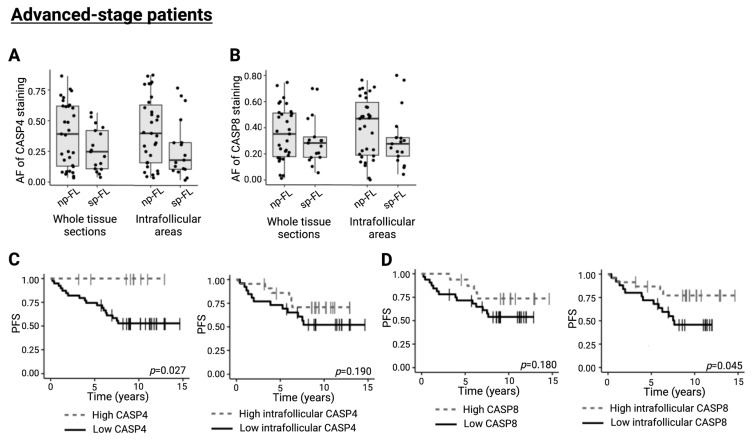
Immunohistochemical evaluation of CASP4 and CASP8 in advanced-stage disease. (**A**) Representative images of immunohistochemical staining patterns of CASP8 expression (left, original magnification ×5; right, original magnification ×20). (**B**) CASP8 expression levels identified by immunohistochemical staining in whole biopsy and intrafollicular areas. (**C**) Association between whole biopsy and intrafollicular CASP4 expression and PFS (cutoffs, AF = 0.6141 and AF = 0.3226, respectively). (**D**) Association between whole biopsy and intrafollicular CASP8 expression and PFS (cutoffs, AF = 0.4703 and AF = 0.3259, respectively). Abbreviations: AF, area fraction; CASP4/8, caspase 4/8; np-FL, non-progressing FL; OS, overall survival; PFS, progression-free survival; and sp-FL, subsequently progressing FL.

Evaluating CASP4 and CASP8 in lymphoma samples from the advanced-stage cohort (*n* = 48) by IHC revealed no significant differential expression (*p* = 0.141 and *p* = 0.310, respectively) (Figure 3A,B). Dichotomizing samples based on CASP4 expression revealed an inferior PFS associated with lower CASP4 expression (*p* = 0.027) (Figure 3C). This was also true for CASP8 when counting intrafollicular areas exclusively (*p* = 0.045) (Figure 3D). In this cohort, the expression levels of CASP4 and CASP8 also showed a significant strong correlation to each other (*p* < 0.001, ρ = 0.53). Altogether, these results indicate the collective impact from several caspase regulators in the disease progression of follicular lymphoma independent of the disease stage at diagnosis.

## 3. Discussion

Employing high-throughput proteomics, we uncovered differential protein expression patterns associated with the risk of disease progression in diagnostic lymphoma samples from limited-stage FL patients. Moreover, our study identified two specific caspase proteins that could serve as potential prognostic biomarkers. Future large-scale and independent studies are warranted to determine whether these findings could be correlated with disease progression and potentially guide clinical decision-making by identifying high-risk patients who might benefit from more intensive treatment, while sparing low-risk patients from unnecessary therapy or enabling less frequent follow-up.

The study was performed as a hypothesis-generating investigation with the aim of identifying novel markers important for disease progression in FL treated with radiation therapy. The focus of the present study was a cohort defined by strict inclusion criteria to ensure that the biology of the lymphoma specimens was as similar as possible and that the results are thus minimally influenced by variations in clinical parameters. As a consequence, the cohort was limited to 26 limited-stage FL patients treated with radiation therapy, which may influence the statistical power of the study and thus prevented the detection of some predictive markers. Still, despite this rather size-limited cohort, our comparative analysis of the protein profiles between sp-FL and np-FL samples did reveal significant differences in protein expression. Most differences in protein expression were rather subtle, with relatively small differences in fold changes despite their significance. This might partly be explained by the ratio compression from the TMT labeling method, for which quantitative differences between samples may be underestimated. Nevertheless, based on these identified protein profiles, we were able to show the discrimination of samples depending on disease outcome. Importantly, both the MS-based and IHC-based analyses identify protein expression levels and thus do not provide direct evidence of the biological roles of these proteins in FL pathogenesis. Despite ongoing research in the field of FL, the mechanisms underlying disease progression remain poorly understood. Thus, our study advances this understanding by offering evidence at the protein expression level. This is especially relevant in the case of limited-stage FL patients treated with radiation therapy. While generally considered incurable, radiation therapy does in some cases provide disease control and might even be administered with curative intent [17,18]. Identifying patients at a higher or lower risk of disease progression, respectively, may therefore be valuable clinically and when guiding therapeutic decision-making.

We identified several deregulated proteins which might contribute to the progression of disease in FL. Notably, these included a network of downregulated proteins including CASP4, CASP8, JAK1, and MAPK14, which were all involved as crucial components of the innate immune system [19]. The two caspases were then further evaluated in lymphoma tissues by IHC. Caspase proteins are a family of proteases and key regulators of inflammation and apoptosis [20]. Expression of CASP4 by IHC showed a significant downregulation in sp-FL compared with np-FL samples. This observation was also trending when analyzing exclusively intrafollicular areas, suggesting that the malignant cells of sp-FL samples express lower levels of CASP4 compared with the np-FL samples. Moreover, a low CASP4 expression also correlated to inferior PFS. While other previous studies have reported high CASP4 expression as a marker for poor prognosis in several cancers, downregulation of CASP4 may play a role in progression of the disease, as it allows for immune evasion and thus sustained growth [21,22,23]. Supportive of this was the association of lower CASP4 expression to inferior PFS in a cohort of advanced-stage FL patients. In limited-stage FL, CASP8 was also found to be downregulated in sp-FL samples compared with np-FL. CASP8 is known as an initiator protease of the extrinsic pathway of apoptosis^22^. However, studies have found that the downregulation of CASP8 may lead to an increased susceptibility to necroptosis, an alternative cell death pathway [24]. When evaluated with IHC, differences in the CASP8 expression between sp-FL and np-FL samples did not reach a statistical difference. However, when dichotomized based on CASP8, a low expression was associated with inferior PFS in limited-stage FL, which was also observed for intrafollicular expression in advanced-stage FLs. In line with this, we previously identified a third caspase protein, CASP3, to be strongly associated with the histological transformation of FL in an independent patient cohort [3]. Interestingly, joint low expression of CASP4 and CASP8 was present in 4 sp-FL samples and may merit a collaborative effect of the two caspase proteins. Together, these findings suggest a dysregulation of caspase proteins in the disease progression of FL, which merits further research into the caspase protein family and apoptotic regulation in the context of FL.

Although further evaluation by IHC did not reach the same level of significance as MS-based proteomics, trends were observed. Several methodological factors may underlie such inconsistencies between the two techniques [16]. Importantly, the proteomic analysis was performed on bulk tissue sections including both lymphoid and non-lymphoid tissue, while the IHC evaluation was restricted to the defined ROI. As such, proteomics was performed as an explorative hypothesis-generating method, while relevant proteins were further investigated using the more clinically implementable IHC methodology. Moreover, IHC allows for the investigation of proteins in their tissue surroundings, thereby providing a perspective on both the tumor cells and non-malignant cells in the TME. The selected proteins, CASP4 and CASP8, did show correlated associations to disease progression; however, alone, they are probably not the main drivers of prognosis in limited-stage FL. Future independent investigations of prognoses in limited-stage FL are warranted to elucidate the possible importance of the caspase protein family in FL.

Another network of proteins centered around metabolism includes the differential expression of proteins TPI1 and ENO1. Both proteins are involved in glycolysis and were found downregulated in sp-FL samples. This observation is in contrast with the fact that glycolysis is commonly upregulated in cancers, and especially aggressive cancers that are known to have higher metabolic rates because of their high turnover [25]. Similarly, our group previously performed a comparable MS-based proteomics study in a size-limited cohort of FL, comparing groups with histological transformation as the endpoint [26]. This study also identified several glycolytic markers to be downregulated in samples from patients with subsequent transformation compared with those without, including aldolase A (ALDOA) and glyceraldehyde-3-phosphate dehydrogenase (GAPDH). However, when evaluated by immunohistochemistry, the inverse was observed, with subsequently transforming samples expressing higher levels of both ALDOA and GAPDH compared with non-transforming samples [26,27]. The MS-based proteomics provides expression levels inferred from the average of all cells within the tissue section; thus, findings are likely to be different between the non-malignant TME and malignant B cells. These results suggest a metabolic alteration in the sp-FL samples, which might contribute to the subsequent progression of disease; this suggests that further investigation into metabolic processes within disease progression in FL is warranted.

Progression of FL disease—especially the early progression of disease within 24 months from treatment initiation (POD24)—has, in recent years, become a clinically relevant endpoint [11,28]. Comprehensive profiling of tumoral components, unveiling the crosstalk between malignant and microenvironmental cells, is warranted to elucidate and identify robust biomarkers of high-risk FL populations, as well as identify potential new treatment targets [15,29].

## 4. Materials and Methods

### 4.1. Patients

All analyses were performed on formalin-fixed, paraffin-embedded (FFPE) diagnostic lymphoma samples from a cohort consisting of 26 FL patients diagnosed with limited-stage FL, grade 1–2, at Aarhus University Hospital between 2003 and 2014. All patients were subsequently treated with radiation therapy. From these patients, 17 did not experience disease progression for at least five years after treatment initiation (non-progressing FL, np-FL), while 9 patients presented with histologically confirmed subsequent disease progression (subsequently progressing FL, sp-FL). We defined the time of progression as the date second-line therapy was initiated. All samples from diagnosis and progression were histologically reviewed by an experienced hematopathologist, according to the 2022/2024 WHO criteria [1]. Clinicopathological data for all patients were collected from the Danish Lymphoma Registry [30]. Patients included for analysis were selected carefully based on several criteria: limited-stage (Ann Arbor I–II), grade 1–2, no composite histology (i.e., no component of large B-cell lymphoma), and no prior treatment [12]. The study period spanned from 2003 until last follow-up in 2023, Figure S1.

The study was approved by the National Committee on Health Research Ethics in Denmark (2007991) and the Danish Data Protection Agency (1-16-02-237-20) and was conducted in compliance with the principles of the Helsinki Declaration.

### 4.2. Identification of Differentially Expressed Proteins

To identify differentially expressed proteins between np-FL and sp-FL samples, a proteomics analysis utilizing tandem mass tag (TMT) labeled nano-liquid chromatography tandem MS (nLC-MS/MS) was conducted, as previously described [3,16,31,32,33,34,35,36,37,38]. The bioinformatic evaluation was carried out by using the Search Tool for the Retrieval of Interacting Genes/Proteins (STRING) database [36,37,38].

The expression levels of two selected proteins, CASP4 and CASP8, identified through the proteomics approach, were further evaluated using IHC. Detailed protocols are provided in the Supplementary Methods.

### 4.3. Statistical Analyses

Differences in clinicopathological features were evaluated using either χ^2^-test or Fisher’s exact test as appropriate. To assess fold changes in differentially expressed proteins between np-FL and sp-FL samples, a Student’s t-test was utilized. For principal component analysis (PCA), only proteins without missing values across patients were included to avoid data imputation. Hierarchical clustering was conducted using Euclidian distance to measure dissimilarity and Ward’s method for cluster linkages. Differences in immunohistochemical AFs between np-FL and sp-FL samples were calculated using an independent Mann–Whitney U test. Spearman’s rank test was used to investigate the correlation between biomarker expression and clinicopathological features. The relatively small sample size of the cohort does not allow for robust multivariate analyses. Receiver operating characteristics (ROCs) curve analysis, with optimal cutoff points determined by Youden’s index, was employed to define high versus low biomarker expression and to analyze time-dependent outcomes. Time-dependent outcomes were assessed using Kaplan–Meier and log-rank methods. Overall survival (OS) was defined as time from initial FL diagnosis to the time of death, while progression-free survival (PFS) was defined as the time from initial FL diagnosis the time of disease progression [3,16]. *p*-values below 0.05 were considered statistically significant. All statistical analyses were conducted using RStudio (version 4.25).

## 5. Conclusions

We performed an explorative study of progression in limited-stage FL. Our data suggests that several biological pathways might be connected to progression, including both alterations of the innate immune system and cellular metabolism. With the present study being explorative in its nature, further studies should focus on validating the presented results and investigate in greater detail the role and function of these proteins in the context of FL. Currently, FL patients exhibit varying clinical outcomes, with disease progression serving as a crucial endpoint. Accurately identifying patients at the time of FL diagnosis who are at a higher risk of progression would represent a significant clinical advancement.

## Figures and Tables

**Table 1:** Patient clinicopathological data.

	Total, *n* = 26 *n* (%)	sp-FL, *n* = 9 *n* (%)	np-FL, *n* = 17 *n* (%)	*p*-Value
Sex				NS
Male	10 (38)	5 (56)	5 (29)	
Female	16 (62)	4 (44)	12 (71)	
Age at diagnosis, y				NS
Median	69	71	69	
Range	23–86	34–83	23–86	
FL grade				NS
1	11 (42)	5 (56)	6 (35)	
1/2	4 (16)	0 (0)	4 (24)	
2	11 (42)	4 (44)	7 (41)	
Ann Arbor Stage				NS
I	24 (92)	9 (100)	15 (88)	
II	2 (8)	0 (0)	2 (12)	
B-symptoms				NS
No	26 (100)	9 (100)	17 (100)	
Yes	0 (100)	0 (0)	0 (0)	
Bulky disease				
No	20 (77)	6 (67)	14 (82)	NS
Yes	1 (4)	1 (11)	0 (0)	
Unknown	5 (19)	2 (22)	3 (18)	
LDH-elevation				
No	22 (85)	7 (78)	15 (88)	NS
Yes	3 (12)	1 (11)	2 (12)	
Unknown	1 (4)	1 (11)	0 (0)	
FLIPI				
Low	23 (88)	7 (78)	16 (94)	NS
Intermediate	2 (18)	1 (11)	1 (6)	
Unknown	1 (4)	1 (11)	0 (0)	

Abbreviations: FLIPI, follicular lymphoma international prognostic index; LDH, lactate dehydrogenase; np-FL, non-progressing FL; NS, not significant; sp-FL, subsequently progressing FL; and y, years.

## Data Availability

Data included in the current study are available upon reasonable request to the corresponding authors.

## Supplementary Materials

The following supporting information can be downloaded at: https://www.mdpi.com/article/10.3390/ijms26199306/s1.
