# Supplementary material for: Proteomic Profiling of Limited-Stage Follicular Lymphoma Reveals Differentially Expressed Proteins Linked to Disease Progression Post-Radiation Therapy
Source: Int J Mol Sci. 2025 Sep 23;26(19):9306. doi: 10.3390/ijms26199306 (PMC12525013; doi:10.3390/ijms26199306)
Supplement: Supplementary file 1 [file ijms-26-09306-s001.zip › ijms-3796422-supplementary.pdf]

Supplementary methods

Sample preparation for MS-based proteomics

A total of 26 diagnostic FFPE lymphoma samples from patients diagnosed with limited-stage FL (i.e., 9 sp-FL and 17 np-FL) were analyzed by tandem mass tag (TMT) mass spectrometry (MS)-based proteomics. Seven 10 µm sections of FFPE tissue from each sample were prepared for protein analysis essentially as previously described[1-3]. Paraffin was removed with xylene followed by rehydration with decreasing levels of ethanol/water, then dried and dissolved in lysis buffer (5% SDS, 50 mM triethylammonium bicarbonate (TEAB), pH 8.5). The protein concentration was estimated using infrared spectrometry (Direct Detect Spectrometer, Merck KGaA, Darmstadt, Germany)[3].

Sample preparation was performed with the suspension-trapping method[4] using S-TrapTM micro columns (Protifi, Farmingdale, NY, USA), as previously described[5]. Samples were processed for labeling with TMT 10plexTM isobaric mass tagging kit (Thermo Fisher Scientific, Waltham, MA, USA) in ten groups as described in detail. A standard sample was prepared by taking 3 µg peptide from each of 26 samples[2].

Quantification with Tandem Mass Tag-Based Mass Spectrometry

A total of 0.5 µg of each fraction was injected into a Dionex Ultimate 3000 RSCL nano LC-system connected to an Orbitrap Fusion Tribrid mass spectrometer (Thermo Fisher Scientific Instruments). Liquid chromatography and MS were performed using the TMT synchronous precursor selection MS^3 method, with settings as described previously[2].

The raw files from the MS analysis were entered and further analyzed in MaxQuant (MaxQuant version 1.6.3.4, Max Planck Institute of Biochemistry, Martinsried, Germany:

https://maxquant.net/maxquant/)[6] using the UniProt Homo sapiens filtered and reviewed database (www.uniprot.org), downloaded March 9th 2021. The generated protein-groups file was entered into Perseus version 1.6.14.0 (Max
Planck Institute of Biochemistry: https://maxquant.net/perseus/)[7]. The proteins were identified based on at least two unique peptides and the normalized set of log_2 transformed reporter values for the 26 samples, without the standards, were then used for further analyses[2]. P-values were calculated by two-tailed t-test without further correction in order to not increase type 2 errors with the risk of overlooking putative predictive markers.

Bioinformatic analysis

Bioinformatic analysis was performed using the STRING database (string-db.org). Each protein's corresponding UniProt ID were submitted to the software. If multiple UniProt IDs were identified, only one, the first listed, was used for the analysis. All 78 significantly differentially expressed proteins identified were entered into the STRING software tool and analyzed. The database was assessed using the String App (version 1.7.0) in Cytoscape (version 3.9.1)[8-10]. The minimum required interaction was set to confidence = 0.2, including both functional and physical interactions. On the entire network of proteins, enrichment analysis was performed. Here, only terms from Gene Ontology (GO) biological processes, GO molecular functions, Kyoto Encyclopedia of Genes and Genomes pathways (KEGG), Reactome pathways, and WikiPathways. The false discovery rate was set to 5%. The option to remove redundant terms was enabled with the redundancy cut-off set to 0.5[2].

Immunohistochemical staining of selected proteins

From the MS-based proteomics, two proteins, CASP4 and CASP8, were selected for evaluation using IHC. Immunohistochemical staining was performed on 4 μm FFPE sections from whole tissue using the Ventana Benchmark Ultra automated staining system (Ventana Medical Systems, Roche, Oro Valley, AZ, USA) as previously described[1,2]. Slides were deparaffinized with EZ Prep solution (Ventana, 950-102), followed by blocking of endogenous peroxidase activity using the OptiView DAB IHC Detection Kit (Ventana, 760-700)[1,2]. Heat induced epitope retrieval (HIER) for both proteins was performed by heating slides to 100°C in ULTRA Cell Conditioning Solution 1 (CC1, Ventana, 950-224) for 64 minutes. Next, primary antibody (CASP4, HPA027588, Ventana; CASP8, HPA001302, Ventana) was diluted to optimal dilution (CASP4 1:400; CASP8 1:100) in REALTM antibody diluent (Dako, S202230-2) and added to the tissue and incubated at 37°C for 32 minutes. Visualization was done using the OptiView IHC DAB Detection Kit (Ventana, 760-700) with nuclear counterstaining by hematoxylin. Sections of appendix, tonsil liver, and pancreas were included on all slides as controls[1,2].

Digital image analysis

All stained slides were scanned on the NanoZoomer 2.0HT (Hamamatsu, Shizouka, Japan) at a magnification of 20x. The scanned images were analyzed using the Visiopharm 2020.08 system (Visiopharm A/S Hoersholm, Denmark). Here, areas for staining quantification were defined by manual outlining of regions of interest (ROI) on each digitized whole tissue section. Here, areas of non-lymphoid tissue and technical artefacts were excluded. Analysis protocol packages were then designed to quantify the expression levels of each marker, as previously described[1,2]. Quantification results from staining were expressed as area fractions (AFs), calculated as the ratio of the stained area to the overall area within the ROI[1,2].

References

1. Enemark MBH, Wolter K, Campbell AJ, et al. Proteomics identifies apoptotic markers as predictors of histological transformation in patients with follicular lymphoma. *Blood Adv*. Dec 26 2023;7(24):7418-7432. doi:10.1182/bloodadvances.2023011299
2. Hemmingsen JK, Enemark MH, Sørensen EF, et al. Proteomic Profiling Identifies Predictive Signatures for Progression Risk in Patients with Advanced-Stage Follicular Lymphoma. *Cancers (Basel)*. Sep 26 2024;16(19)doi:10.3390/cancers16193278
3. Honoré B. Proteomic Protocols for Differential Protein Expression Analyses. *Methods Mol Biol*. 2020;2110:47-58. doi:10.1007/978-1-0716-0255-3_3
4. Zougman A, Selby PJ, Banks RE. Suspension trapping (STrap) sample preparation method for bottom-up proteomics analysis. *Proteomics*. May 2014;14(9):1006-0. doi:10.1002/pmic.201300553
5. Cehofski LJ, Kojima K, Terao N, et al. Aqueous Fibronectin Correlates With Severity of Macular Edema and Visual Acuity in Patients With Branch Retinal Vein Occlusion: A Proteome Study. *Invest Ophthalmol Vis Sci*. Dec 1 2020;61(14):6. doi:10.1167/iovs.61.14.6
6. Tyanova S, Temu T, Cox J. The MaxQuant computational platform for mass spectrometry-based shotgun proteomics. *Nat Protoc*. Dec 2016;11(12):2301-2319. doi:10.1038/nprot.2016.136
7. Tyanova S, Temu T, Sinitcyn P, et al. The Perseus computational platform for comprehensive analysis of (prote)omics data. *Nat Methods*. Sep 2016;13(9):731-40. doi:10.1038/nmeth.3901
8. Doncheva NT, Morris JH, Gorodkin J, Jensen LJ. Cytoscape StringApp: Network Analysis and Visualization of Proteomics Data. *J Proteome Res*. Feb 1 2019;18(2):623-632. doi:10.1021/acs.jproteome.8b00702
9. Szklarczyk D, Gable AL, Lyon D, et al. STRING v11: protein-protein association networks with increased coverage, supporting functional discovery in genome-wide experimental datasets. *Nucleic Acids Res*. Jan 8 2019;47(D1):D607-d613. doi:10.1093/nar/gky1131
10. Szklarczyk D, Gable AL, Nastou KC, et al. The STRING database in 2021: customizable protein-protein networks, and functional characterization of user-uploaded gene/measurement sets. *Nucleic Acids Res*. Jan 8 2021;49(D1):D605-d612. doi:10.1093/nar/gkaa1074

Supplementary tables

Table S1: Significantly differentially expressed proteins identified between sp-FL and np-FL samples

Fold changes (sp-FL/np-FL)	p-value	Gene name	Protein name
**Upregulated**			
0.49	0.047	*GSTM2*	Glutathione S-transferase Mu 2
**Downregulated**			
-0.21	0.038	*GARS*	Glycine--tRNA ligase
-0.25	0.036	*ARHGAP1*	Rho GTPase-activating protein 1
-0.26	0.033	*FAM65B*	Protein FAM65B
-0.27	0.045	*SMAP2*	Stromal membrane-associated protein 2
-0.28	0.018	*ENO1*	Alpha-enolase
-0.28	0.047	*SFN*	14-3-3 protein sigma
-0.29	0.022	*EIF3K*	Eukaryotic translation initiation factor 3 subunit K
-0.29	0.033	*EVL*	Ena/VASP-like protein
-0.31	0.037	*ACAT2*	Acetyl-CoA acetyltransferase, cytosolic
-0.31	0.042	*EIF3H*	Eukaryotic translation initiation factor 3 subunit H
-0.32	0.021	*DARS*	Aspartate--tRNA ligase, cytoplasmic
-0.32	0.022	*PGM1*	Phosphoglucomutase-1
-0.33	0.020	*EEF1E1*	Eukaryotic translation elongation factor 1 epsilon-1
-0.33	0.047	*NFATC2*	Nuclear factor of activated T-cells, cytoplasmic 2
-0.34	0.025	*YWHAG*	14-3-3 protein gamma
-0.35	0.048	*STK39*	STE20/SPS1-related proline-alanine-rich protein kinase
-0.37	0.010	*YWHAQ*	14-3-3 protein theta
-0.37	0.031	*TPI1*	Triosephosphate isomerase
-0.37	0.032	*FASN*	Fatty acid synthase
-0.37	0.042	*EEF1B2*	Elongation factor 1-beta
-0.41	0.024	*ELMO1*	Engulfment and cell motility protein 1
-0.41	0.033	*UQCRC2*	Cytochrome b-c1 complex subunit 2, mitochondrial
-0.41	0.043	*TBL1XR1*	F-box-like/WD repeat-containing protein TBL1XR1
-0.42	0.005	*SCPEP1*	Retinoid-inducible serine carboxypeptidase
-0.42	0.010	*GART*	Trifunctional purine biosynthetic protein adenosine-3
-0.42	0.036	*M6PR*	Cation-dependent mannose-6-phosphate receptor
-0.42	0.037	*PPIL3*	Peptidyl-prolyl cis-trans isomerase-like 3
-0.43	0.027	*DHX30*	Putative ATP-dependent RNA helicase DHX30
-0.43	0.031	*BPNT1*	3(2),5-bisphosphate nucleotidase 1
-0.44	0.031	*PDAP1*	28 kDa heat- and acid-stable phosphoprotein
-0.45	0.017	*STX7*	Syntaxin-7
-0.45	0.022	*PFKP*	ATP-dependent 6-phosphofructokinase, platelet type
-0.45	0.033	*HEXA*	Beta-hexosaminidase subunit alpha
-0.46	0.013	*IRF8*	Interferon regulatory factor 8
-0.47	0.024	*NAP1L4*	Nucleosome assembly protein 1-like 4
-0.47	0.040	*ECH1*	Delta(3,5)-Delta(2,4)-dienoyl-CoA isomerase, mitochondrial
-0.48	0.024	*ATP5B*	ATP synthase subunit beta, mitochondrial
-0.48	0.033	*GBP2*	Interferon-induced guanylate-binding protein 2

-0.48	0.034	*CASP4*	Caspase-4
-0.49	0.032	*ATP5C1*	ATP synthase subunit gamma, mitochondrial
-0.53	0.021	*JAK1*	Tyrosine-protein kinase JAK1
-0.53	0.045	*TPP1*	Tripeptidyl-peptidase 1
-0.54	0.018	*CASP8*	Caspase-8
-0.54	0.049	*HVCN1*	Voltage-gated hydrogen channel 1
-0.57	0.003	*WARS*	Tryptophan--tRNA ligase, cytoplasmic
-0.57	0.037	*SLC25A11*	Mitochondrial 2-oxoglutarate/malate carrier protein
-0.58	0.023	*TOMM40*	Mitochondrial import receptor subunit TOM40 homolog
-0.58	0.025	*MAPK14*	Mitogen-activated protein kinase 14
-0.58	0.028	*SNX29P2*	Putative protein SNX29P2
-0.58	0.043	*NACA*	Nascent polypeptide-associated complex subunit alpha
-0.59	0.037	*AP1M1*	AP-1 complex subunit mu-1
-0.59	0.045	*SELT*	Selenoprotein T
-0.60	0.018	*BTF3*	Transcription factor BTF3
-0.60	0.023	*BCAP31*	B-cell receptor-associated protein 31
-0.60	0.032	*YKT6*	Synaptobrevin homolog YKT6
-0.60	0.040	*RPL30*	60S ribosomal protein L30
-0.61	0.035	*MRPL11*	39S ribosomal protein L11, mitochondrial
-0.62	0.037	*VDAC2*	Voltage-dependent anion-selective channel protein 2
-0.63	0.036	*UBAP2L*	Ubiquitin-associated protein 2-like
-0.64	0.040	*SUPT5H*	Transcription elongation factor SPT5
-0.65	0.037	*EEFSEC*	Selenocysteine-specific elongation factor
-0.66	0.015	*MRPL49*	39S ribosomal protein L49, mitochondrial
-0.66	0.022	*C1QBP*	Complement component 1 Q subcomponent-binding protein, mitochondrial
-0.66	0.023	*VDAC1*	Voltage-dependent anion-selective channel protein 1
-0.67	0.020	*GAPVD1*	GTPase-activating protein and VPS9 domain-containing protein 1
-0.71	0.025	*ADK*	Adenosine kinase
-0.73	0.015	*ACADM*	Medium-chain specific acyl-CoA dehydrogenase, mitochondrial
-0.77	0.032	*RAB1B*	Ras-related protein Rab-1B
-0.77	0.038	*SLC25A5*	ADP/ATP translocase 2
-0.79	0.035	*IMPDH1*	Inosine-5-monophosphate dehydrogenase 1
-0.85	0.010	*RAE1*	mRNA export factor
-0.86	0.031	*MIOS*	WD repeat-containing protein mio
-0.89	0.033	*CD2BP2*	CD2 antigen cytoplasmic tail-binding protein 2
-0.90	0.029	*TSSC1*	Protein TSSC1
-0.92	0.042	*UQCRC1*	Cytochrome b-c1 complex subunit 1, mitochondrial
-0.94	0.010	*ASAP1*	Arf-GAP with SH3 domain, ANK repeat and PH domain-containing protein 1
-1.01	0.006	*GLB1*	Beta-galactosidase

Abbreviations: np-FL, non-progressing FL; sp-FL, subsequently-progressing FL.

Table S2: Pathway analysis based on significantly differentially expressed proteins

Category	Associated function	FDR value	p-value	No. of proteins
Reactome Pathways	Metabolism	0.0000855	3.75E-08	26
GO Molecular Function	RNA binding	0.0011	0.000000227	22
KEGG Pathways	NOD-like receptor signaling pathway	0.0018	0.00000593	7
KEGG Pathways	Prion disease	0.0022	0.00000945	8
Reactome Pathways	Intrinsic Pathway for Apoptosis	0.0025	0.00000263	5
Reactome Pathways	Translation	0.0025	0.00000215	9
KEGG Pathways	Lysosome	0.0061	0.00014	5
Reactome Pathways	Infectious disease	0.0061	0.0000107	14
GO Molecular Function	Porin activity	0.0074	0.00000301	3
Reactome Pathways	Glucose metabolism	0.0081	0.0000342	5
GO Molecular Function	Binding	0.0084	0.00000509	67
KEGG Pathways	Cellular senescence	0.0112	0.00033	5
KEGG Pathways	Hepatitis B	0.0126	0.00041	5
KEGG Pathways	Influenza A	0.0133	0.00047	5
WikiPathways	Clear cell renal cell carcinoma pathways	0.0166	0.0000212	5
Reactome Pathways	Cytosolic tRNA aminoacylation	0.0227	0.00015	3
KEGG Pathways	Salmonella infection	0.0337	0.0014	5
KEGG Pathways	Glycosphingolipid biosynthesis - ganglio series	0.0352	0.0017	2

Abbreviations: FDR, false discovery rate; GO, gene oncology; KEGG, Kyoto Encyclopedia of Genes and Genomes.

Supplementary figures

Figure S1

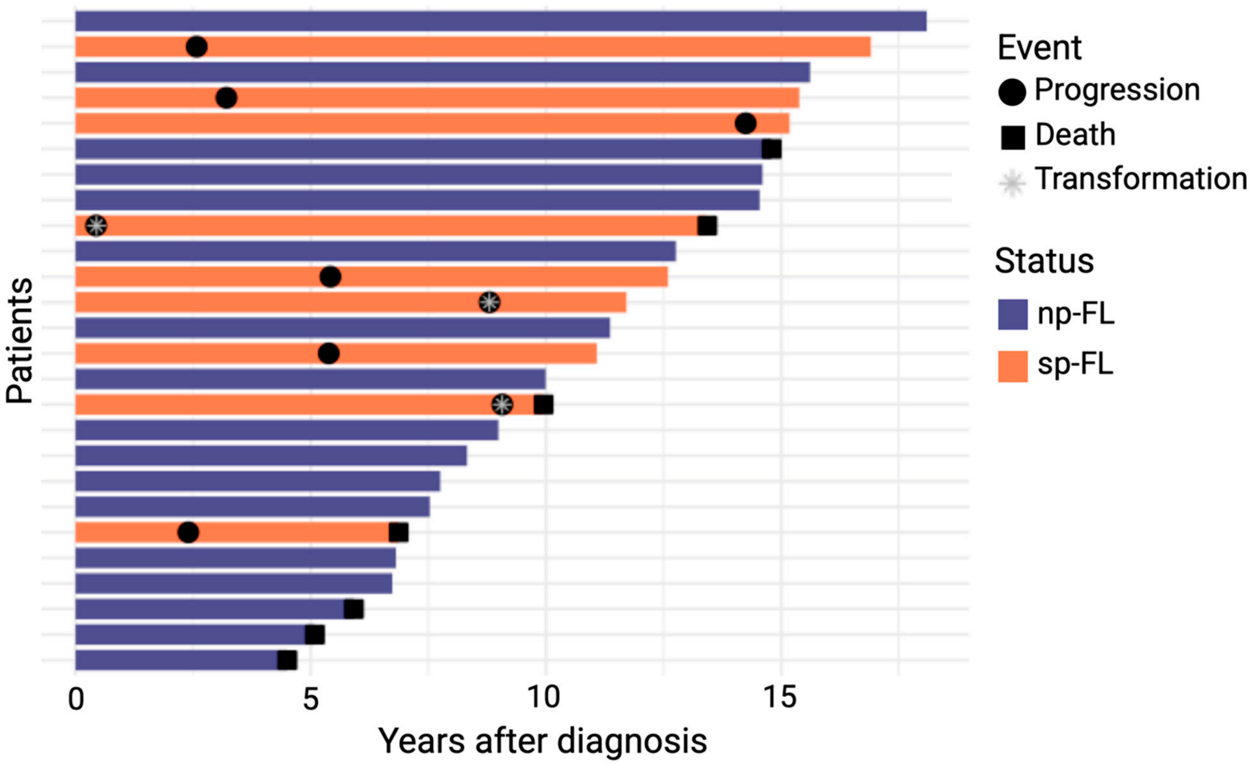

Figure S1: Progression, transformation, and death in the patient cohort.
Swimmer plot depicting follow up times as well as time of progression, transformation, and death for all included limited-stage patients. Abbreviations: np-FL, non-progressing FL; sp-FL, subsequently-progressing FL.

Figure S2

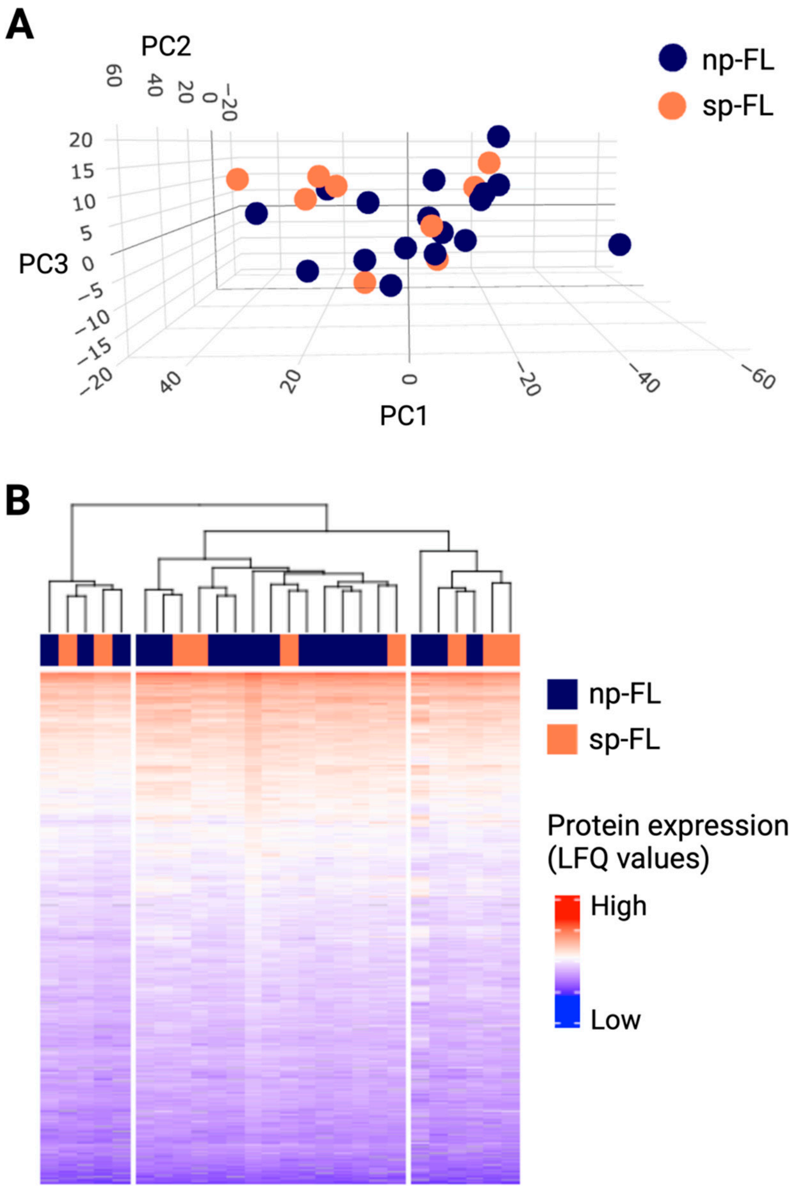

Figure S2: Unsupervised clustering based on all identified proteins.
(A-B) 3D PCA and hierarchal clustering based on all 1940 identified proteins comparing np-FL and sp-FL samples. Abbreviations: PC, principal component; LFQ, label-free quantification; np-FL, non-progressing FL; sp-FL, subsequently-progressing FL.

Figure S3

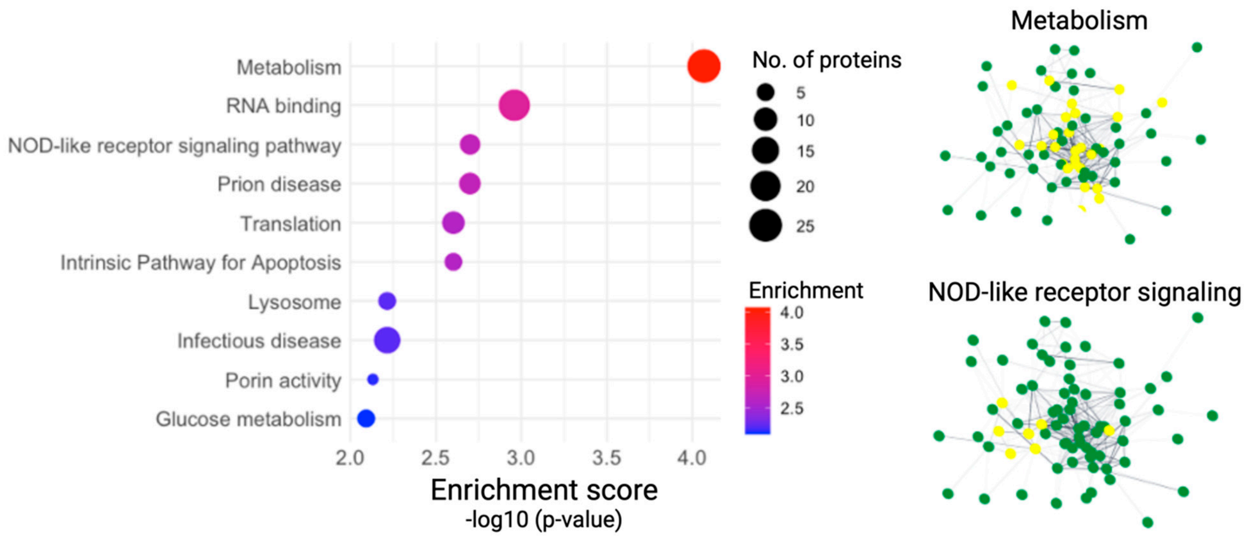

Figure S3: Pathway analysis based on the significantly differentially expressed proteins. Right: Enrichment analysis based on 78 identified significantly differentially expressed proteins. Increasing sizes indicate more involved proteins, with color corresponding to the enrichment score. Left: Protein-protein interaction network of the 78 proteins. Nodes represent proteins while edges visualize interactions, either functional or physical. Yellow indicates proteins involved in said pathway.
